# Using new satellite based exposure methods to study the association between pregnancy pm_2.5_ exposure, premature birth and birth weight in Massachusetts

**DOI:** 10.1186/1476-069X-11-40

**Published:** 2012-06-18

**Authors:** Itai Kloog, Steven J Melly, William L Ridgway, Brent A Coull, Joel Schwartz

**Affiliations:** 1 Department of Environmental Health - Exposure, Epidemiology and Risk Program, Harvard School of Public Health, Landmark Center 401 Park Dr West, Boston, MA, 02215, USA; 2 Science Systems and Applications, Inc, 10210 Greenbelt Road, Suite 600, Lanham, MD, 20771, USA; 3 Department of Biostatistics, Harvard School of Public Health, Boston, MA, 02215, USA

**Keywords:** Air pollution, Birth weight, Preterm birth, Aerosol optical depth, Epidemiology, PM_2.5_

## Abstract

**Background:**

Adverse birth outcomes such as low birth weight and premature birth have been previously linked with exposure to ambient air pollution. Most studies relied on a limited number of monitors in the region of interest, which can introduce exposure error or restrict the analysis to persons living near a monitor, which reduces sample size and generalizability and may create selection bias.

**Methods:**

We evaluated the relationship between premature birth and birth weight with exposure to ambient particulate matter (PM_2.5_) levels during pregnancy in Massachusetts for a 9-year period (2000–2008). Building on a novel method we developed for predicting daily PM_2.5_ at the spatial resolution of a 10x10km grid across New-England, we estimated the average exposure during 30 and 90 days prior to birth as well as the full pregnancy period for each mother. We used linear and logistic mixed models to estimate the association between PM_2.5_ exposure and birth weight (among full term births) and PM_2.5_ exposure and preterm birth adjusting for infant sex, maternal age, maternal race, mean income, maternal education level, prenatal care, gestational age, maternal smoking, percent of open space near mothers residence, average traffic density and mothers health.

**Results:**

Birth weight was negatively associated with PM_2.5_ across all tested periods. For example, a 10 μg/m^3^ increase of PM_2.5_ exposure during the entire pregnancy was significantly associated with a decrease of 13.80 g [95% confidence interval (CI) = −21.10, -6.05] in birth weight after controlling for other factors, including traffic exposure. The odds ratio for a premature birth was 1.06 (95% confidence interval (CI) = 1.01–1.13) for each 10 μg/m^3^ increase of PM_2.5_ exposure during the entire pregnancy period.

**Conclusions:**

The presented study suggests that exposure to PM_2.5_ during the last month of pregnancy contributes to risks for lower birth weight and preterm birth in infants.

## Background

Recent epidemiological studies have established the association between maternal exposure to air pollution and adverse pregnancy outcomes [1-10]. The studies have shown that exposure to air pollution may elevate the risk of adverse birth outcomes, including infant death [11],low birth weight (LBW) [4,12,13] , preterm delivery [8], and small body size for gestational age [14].

Low or reduced birth weight (LBW) is an important predictor of children’s health and is associated with higher risk of infant and childhood mortality [15] and coronary heart disease [16]. Preterm birth (PTB) is an indicator of prenatal disturbances of the placenta and of fetal development. Like LBW, prematurity is an important predictor of infant mortality, childhood morbidity, and possibly adult morbidity [8].

Interpretations of studies are complicated since LBW (birth weight smaller than 2500 g) represents a heterogeneous group of outcomes with different pathogenic mechanisms. Some infants have LBW as a result of PTB (less than 37 completed weeks of gestation at delivery), while others are a result of intrauterine growth restriction- IUGR (birth weight less than that expected for a given gestational age). Some maternal prenatal determinants may be associated with an increased risk of LBW through effects on the length of gestation alone (e.g., premature rupture of the membranes, placenta abruption), others through effects on intrauterine fetal growth alone (e.g., maternal weight gain, hypertension), and some possibly through effects on both PTB and IUGR (e.g., maternal cigarette smoking). Where air pollution falls in this spectrum is not fully resolved.

Previous studies examining the association of LBW/PTB and PM_2.5_ have typically used available monitors in the study area. PM_2.5_ concentrations vary spatially within the study domain and thus using only available monitors introduces exposure error and likely biases the effect estimates downward [17]. Furthermore, lack of spatially resolved daily PM_2.5_ concentration data restricts these studies to populations surrounding monitoring sites, which may not be representative of the population as a whole. Land use regressions provide estimates of geographically resolved exposures at individual residences, but are usually not temporally resolved enough [18,19] to look at effects of exposures in the last 30 days of pregnancy, when a substantial weight gain occurs.

We developed a method to predict daily temporally and spatially resolved PM_2.5_ across New-England for the years 2000–2008 [20,21]. These predictions, which are based on land use regression plus a daily calibration of PM_2.5_ ground measurements and MODIS (Moderate Resolution Imaging Spectroradiometer) satellite aerosol optical depth (AOD), allow us to predict daily PM_2.5_ concentration levels at the resolution of a 10x10 km spatial grid. Recently, this model has been slightly updated to include nested regions in the yearly models and weights to account for non-random missingness in AOD. The new “out of sample” R^2^ of the prediction model is 0.85. Importantly, this R^2^ is for daily observations, rather than monthly or yearly values. By averaging our estimated daily exposures at each location we can generated long term exposures. This enables us to study both the short term and long term effects of ambient particles, respectively.

In the presented study, we make use of these new PM_2.5_ prediction data to study the association between long (exposure during the whole birth period and last trimester) and short term PM_2.5_ exposure (exposure during the last month of pregnancy) and birth weight and premature birth in eastern Massachusetts between the years 2000–2008. The resulting analyses include birth weight outcomes from all births in the study region regardless of how close each participant lives to a PM_2.5_ monitor.

## Methods

### Study domain and population

**Figure 1:**
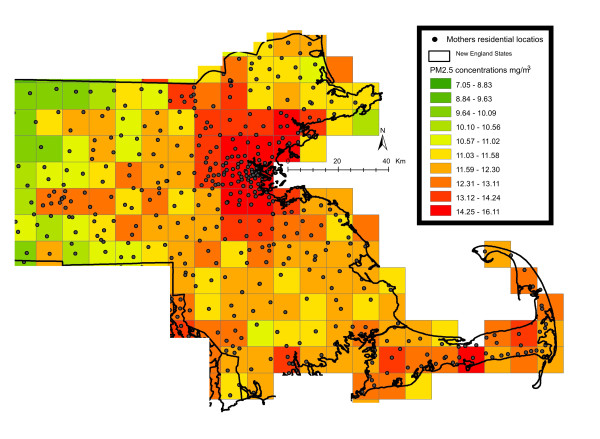
**Map of the study area showing the residential location of a subset of mothers over a sample PM**_**2.5**_**(μg/m3) 10x10km pollution grid for a sample day (01/07/2001).**

The spatial domain of our study included the state of Massachusetts (Figure 1). The study population included all singleton live births in Massachusetts from the Massachusetts Birth Registry for the period of January 1, 2000 to December 31, 2008. The residential address of each mother at time of birth was geocoded by a private firm – Teleatlas, an industry leading geocoding company commonly used in academic studies [22]. In addition, we manually checked a random sample of geocoded addresses for accuracy by using ESRI ArcGIS^©^ software StreetMap data [23]. Geocoding was done by matching the street address, city, state, and ZIP code to street network data derived from US census bureau TIGER (Topologically Integrated Geographic Encoding and Referencing system) dataset [24], and assigning to this street address latitude and longitude coordinates. The study and the use of birth data was approved by the Massachusetts Department of Public Health and the human subjects committee of the Harvard School of Public Health. The analysis was restricted to singleton births, and there were 634,244 such births in the study. The number of infants with gestational age of 37 weeks or greater was 572,272.

### Exposure data

For exposure data we used two different indicators: predicted 10 x 10 km PM_2.5_ and residence-specific cumulative traffic density. We describe each metric in more detail below.

*PM*_*2.5*_*exposure -* PM_2.5_ exposure data were generated by the previously mentioned PM_2.5_ prediction model [20]. In these prediction models we used mixed models with random slopes for day to calibrate satellite AOD data at the resolution of a 10x10 km spatial grid (2000–2008) across New England with monitored PM_2.5_ measurements. We then used a generalized additive mixed model with spatial smoothing to estimate PM_2.5_ in location-day pairs with missing AOD, using regional measured PM_2.5_, AOD values in neighboring cells, and land use. ”Out-of-sample” ten-fold cross-validation was used to quantify the accuracy of our predictions. For days with available AOD data we found high “out-of-sample” R^2^ (mean “out-of-sample” R^2^ = 0.87). For days without AOD values, our model performance was also excellent (mean “out-of-sample” R^2^ = 0.85). To estimate PM_2.5_ exposure, each mother’s residence at the time of delivery was linked to one 10X10km grid point (see Figure 1). Exposure was calculated by averaging daily PM_2.5_ concentrations over the 30 days prior to the delivery date, 90 days prior to the delivery date and the full pregnancy period.

*Cumulative traffic density-* Local traffic emissions are a small part of total PM_2.5_ in Boston (for example in our Massachusetts data, black carbon consists of only 10% of total PM). This allows us to separately consider traffic pollution and PM_2.5_. Road data were obtained from the Massachusetts department of transportation (MassDOT), which conducts traffic counts on selected roads and provided estimates of 2002 traffic on other roads. This dataset included average daily traffic (ADT) as an attribute. The data included automatic and estimated counts based on periodic counts on all other major roads. A 200 m by 200 m grid was carefully constructed to allow 100 m buffers around each grid center point without overlap. These buffers covered the seven counties as previously done in several studies [10]. Normalized cumulative ADT (CADT) was calculated for all road segments within 100 meters around each grid point as: CADT = Σ (ADT *road segment length), and that value was assigned to the respective grid point. Birth addresses were then assigned an average of the estimates of the four grid points around it, using bilinear interpolation.

### Covariates

Covariates were chosen based on previous literature on the potential risk factors associated with low birth weight [9,10] and include both individual and contextual covariates:

*Land use for recreation and conservation-* Land use for recreation and conservation (open space) was downloaded from the office of geographic information Commonwealth of Massachusetts, information technology division MassGIS [25]. The subset of the open space designated for recreation and conservation was intersected with 2000 Census tract boundaries (also downloaded from MassGIS) using ArcGIS^©^ 9.3. The percent of each census tract that was open space was then calculated and assigned to birth addresses belonging to that tract.

*Socioeconomic indicators-* Individual level variables were obtained from the birth registry. Such information included the mother's race, mother’s years of education, and the Kotelchuck index of adequacy of prenatal care utilization (APNCU). APNCU is based on the number and the time of start of mother's prenatal visits [26] was recorded into: inadequate (<50 percent of expected visits used); intermediate (50–79 percent); appropriate (80–109 percent); and appropriate plus (≥110 percent).

*Education* of the mother was categorized as: no high school (≤ 12 years of educational attainment), some high school (≤ 12 years of educational attainment); some college (13–15 years); and college or postgraduate (≥ 16 years).

*Median income*- We obtained data from the United States Census Bureau of 2000 on median household income [27] for each census tract in the study area, and assigned these to births whose address belonged to that tract.

Additionally, individual-level covariates maternal age, gestational age, amount of cigarettes smoked during and before pregnancy, chronic conditions of mother or conditions of pregnancy (lung disease, hypertension, gestational diabetes and non-gestational diabetes), previous occurrence of a preterm birth, whether the mother ever had a previous infant weighing 4000 grams or more and gender of infant were all obtained through the Massachusetts Birth Registry.

### Statistical methods

To identify and measure the significance of factors affecting birth weight two models were performed: a linear mixed regression models using birth weight (among full term births) as the outcome and a logistic mixed regression models using pre term/full term birth as the outcome [9,28]. All modeling was done in R statistical software. Predictors included mean PM_2.5_ exposure prior to delivery (30, 90 and 270 days prior to the delivery date), cigarette use previous to pregnancy, cigarette use during pregnancy, median income, APNCU, cumulative traffic density, percent of open spaces, age of mother, gestational age, chronic conditions of mother or conditions of pregnancy (lung disease, hypertension, gestational diabetes or non-gestational diabetes), previous preterm births, previous infant weighting 4000 grams and gender of infant. A random intercept for census tract was used to capture unmeasured similarities in persons in the same neighborhood.

Specifically we fit the models:

(1) $$BW_{\mathrm{ij}}=\left(\alpha +u_{j}\right)+\beta_{1}PM_{i}+\beta_{2i}X_{2i}+\beta_{3i}X_{31}\ldots +e_{\mathrm{ij}}\left(u_{j}\right)\sim N[0,s_{u}{}^{2}]{,}_{\left(model\;1\right)}$$

(2) $$Logit(Pr\;BW_{\mathrm{ij}}=1|X)=\left(\alpha +u_{j}\right)+\beta_{1}PM_{i}+\beta_{2i}X_{2i}+\beta_{3i}X_{31}\ldots +e_{\mathrm{ij}}\left(u_{j}\right)\sim N[0,s_{u}{}^{2}]{,}_{\left(model\;2\right)}$$

where *BW*_*ij*_ (or *Logit(Pr BW*_*ij*_ *= 1|X) )* is the response (birth weight or full/pre term) for the *i*th subject in census tract *j*, *α* and *u*_*j*_ are the fixed and random (tract specific) intercepts, respectively*,* PM_i_, X_1i_, etc. denote the set of covariates of interest used in the model, e_ij_ is the error term and finally, *σ*_*u*_^* 2 i*^is the variance of the tract random effects, and *e*_*jj*_ *~ N[0,σ*_*e*_^* 2*^*],*. During the analysis, multicollinearity and normality were evaluated, and no issues were uncovered (tolerance > 0.4).

## Results

Descriptive statistics are presented in Table 1. Of the 634,844 live full term births included in our analyses, 49.36% of the births were male, 71.70% were white, only 7.50% had maternal age below 20 and 21.16% of the mothers had more than 15 years of education.

**Table 1:** Descriptive statistics: Live births in eastern Massachusetts, 2000–2008

**Characteristic**	**All births (% Total)**	**Mean Birth Weight (Grams)**	**SD**
**Sex**			
Male	49.36	3262.38	616.86
Female	51.14	3379.14	648.75
**Race**			
White	71.70	3368.2	627.35
African-American	7.99	3165.04	688.52
Hispanic	20.31	3220.94	623.57
**Education (years)**			
≤ 8	2.68	3253.04	597.64
>8-12	33.53	3259.15	635.04
13-15	42.62	3333.61	637.41
≥15	21.16	3375.29	621.90
**Age of mother**			
≤ 20	7.50	3192.03	612.17
20-29	34.72	3299.77	617.60
30-34	32.11	3359.42	634.44
35-39	20.41	3356.59	654.62
>39	5.26	3292.81	692.11

Table 2 contains a summary of the predicted exposures across all grid cells in the analysis. Table 3 presents the results from both the logistic mixed model regressions for all exposure periods and the regression model for all exposure periods. In the linear mixed model analyses a 10 μg/m^3^ increase of PM_2.5_ exposure during the last 30 days prior to delivery was associated with a decrease of 8.80 g [95% confidence interval (CI) = −10.32, -4.44] in birth weight. A 10 μg/m^3^ increase of PM_2.5_ exposure during the last 90 days prior to delivery was associated with a decrease of 9.20 g [95% confidence interval (CI) = −15.00, -3.30] in birth weight. Finally, a 10 μg/m^3^ increase of PM_2.5_ exposure during the entire pregnancy was associated with a decrease of 13.80 g [95% confidence interval (CI) = −21.10, -6.04] in birth weight.

**Table 2:** **Descriptive statistics for PM**_**2.5**_**exposure, Massachusetts, 2000–2002**

**Covariate**	**Mean**	**Median**	**SD**	**Range**	**IQR**	**Q1**	**Q3**	**Days of data available**
**PM**_**2.5**_**exposure (μg/m**^**3**^**)**	9.6	8.4	5.1	159.7	5.3	6.3	11.6	3285
Cumulative traffic density (density*length)	1309	702	2076	36188	1352	258	1611	3285

Note: Q1 and Q3 are quartiles.

**Table 3:** **Odds ratio for premature births and change in birth weight for full term births for each 10 μg/m**^**3**^**increment in PM**_**2.5**_**for various exposure periods**

**Exposure**	**Birth weight Change (in grams) (95% CI)**	**OR (95% CI)**
**Last month (30 days prior to birth)**	−8.80^***^ (−10.32 to −4.44)	1.00 (0.96 to 1.04)
**Last trimester (90 days prior to birth**	−9.20^***^ (−15.00 to −3.30)	0.99 (0.94 to 1.03)
**Entire birth period**	−13.80^***^ (−21.10 to −6.05)	1.06^***^ (1.01 to 1.13)

Note: ^***^ indicates a 0.01 significance level.

The odds ratio for a premature birth was 1.06 [95% confidence interval (CI) = 1.01–1.13] for each 10 μg/m^3^ increase of PM_2.5_ exposure during the full pregnancy. Other exposure periods were non-significant.

In addition, all other covariates acted as expected (see Appendix 1). For example cigarette use both pre pregnancy and during pregnancy also decrease birth weight significantly (Beta = −17.30, CI = −17.95,-16.65 per cigarette per day smoked during pregnancy and Beta = −1.45 g, CI = −1.79,-1.11 per cigarettes per day for smoking pre pregnancy) (see Appendix 1). These results are for the full birth PM_2.5_ model but were essentially identical across all 3 averaging times.

## Appendix 1

Factors affecting birth weight for all singleton live births in Eastern Massachusetts between 2000–2008.

**Table:** 

**Model Covariates**	**Birth Weight**	**Changes in grams (CI 95% Low-95% Hi)**	**Premature birth**	**OR (CI 95% Low-95% Hi)**
**PM**_**2.5**_**exposure for entire birth period**	−1.38	(−2.11;-0.65)	1.01	(0.29;0.40)
Normalized cumulative traffic density (density*length)	−0.04	(−0.09;0.02)	1.00	(1.00;1.01)
**Maternal Age (yr)**	7.82	(7.38;8.27)	0.99	(0.99;1.00)
**Maternal Age**^**2**^	−0.21	(−0.24;-0.19)	1.00	(0.98;0.99)
**Cigarettes per day during Pregnancy**	−17.30	(−17.95;-16.65)	1.02	(1.00;1.01)
**Cigarettes per day before Pregnancy**	−1.45	(−1.79;-1.11)	1.00	(1.02;1.03)
**Median Household Income in tract ($10,000)**	1.58	(0.53;2.64)	0.99	(0.99;1.00)
**Percent of Open Spaces**	0.39	(0.2;0.58)	1.00	(0.99;1.00)
**Gender of infant (males vs females)**	−129.93	(−132.3;-127.55)	0.85	(0.83;0.86)
**Previous birth of infant ? 4000 grams**	477.78	(463.82;491.74)	0.46	(0.39;0.55)
**Gestational diabetes**	49.42	(42.86;55.98)	1.33	(1.27;1.41)
**Hypertension**	−91.30	(−98.29;-84.3)	1.94	(1.84;2.04)
**Lung disease**	−30.01	(−36.91;-23.12)	1.25	(1.18;1.32)
**Non-gestational diabetes**	93.18	(79.22;107.14)	1.98	(1.81;2.18)
**Previous occurrence of preterm birth**	−202.07	(−216.13;-188.01)	3.55	(3.31;3.8)
**APNCU:**				
**Appropriate plus**	194.26	(164.19;224.33)	0.25	(0.22;0.29)
**Appropriate**	160.08	(129.92;190.24)	0.31	(0.27;0.36)
**Intermediate**	145.86	(115.1;176.62)	0.31	(0.27;0.36)
**Inadequate**	147.49	(111.8;183.17)	0.45	(0.38;0.55)
**None**	reference			
**Race:**				
**White**	130.47	(127.02;133.93)	0.84	(0.81;0.86)
**African-American**	3.35	(−2.1;8.81)	1.20	(1.15;1.25)
**Hispanic**	reference			
**Education:**				
**Education (reference- college or postgraduate (> 15 years) ):**	−8.74	(−16.88;-0.6)	1.28	(1.21;1.37)
**No High school (< 8 years)**	−20.11	(−23.58;-16.64)	1.21	(1.17;1.25)
**High school (>8 – 12 years)**	0.13	(−3.25;3.52)	1.10	(1.07;1.14)
**Some college (13 – 15 years)**	reference			
**Year:**				
**2000**	14.98	(9.47;20.5)	0.89	(0.85;0.93)
**2001**	9.42	(3.94;14.91)	0.91	(0.86;0.95)
**2002**	3.49	(−1.86;8.85)	0.92	(0.87;0.96)
**2003**	4.60	(−0.63;9.83)	0.97	(0.92;1.01)
**2004**	−1.59	(−6.76;3.58)	1.01	(0.96;1.05)
**2005**	−11.18	(−16.35;-6.01)	1.03	(0.98;1.08)
**2006**	−12.65	(−17.73;-7.57)	1.05	(1.01;1.09)
**2007**	−7.39	(−12.59;-2.18)	1.07	(1.02;1.12)
**2008**	reference			

## Discussion

We examined the effects of PM_2.5_ exposure on birth outcomes in a study of singleton births in seven Massachusetts counties between 2000 and 2008. Using a model based on satellite remote sensing we were able to assign exposure to all subjects, regardless of the distance between a participant’s residence and the closest PM_2.5_ monitor. We found a consistent effect of exposure to PM_2.5_ on birth weight for infants who were born full term, and an elevated risk of preterm delivery after adjusting for other potential risk factors such as previous and current mother’s health conditions, socioeconomic factors and physical environment risk factors. Importantly, this association remained after controlling for traffic density within 100 meters of the residence and open space. Combined with the fact that the satellite grid is too coarse to capture local effects of high traffic on a nearby road, this suggests that the PM_2.5_ results we report here are due predominantly to non-primary traffic particles, and that the traffic density variable captures the additional impact of traffic pollution. In Massachusetts during these years, such non primary particle exposures were mostly sulfates, from coal burning power plants, and transported secondary organic aerosols, which are generated from a variety of sources including traffic in upwind locations.

A key feature of the presented study compared to previous epidemiologic studies showing associations between air pollution and birth weight [10,29,30] is the exposure assignment. Since our model allows us to predict temporally and spatially resolved PM_2.5_ we can assign daily PM_2.5_ exposure to the entire study population, avoiding selection bias that would yield a non-representative sample. In addition we account for small area measures of potential confounders at a 10x10km spatial resolution, thus minimizing exposure error.

Knowledge of the exact impact of PM_2.5_ on birth weight and its determinants is still very limited. Fine particulate matter may affect birth weight through direct or indirect means. Some studies have shown that PM_2.5_ is associated with a number of cardiovascular and respiratory related outcomes both in adults and children [31-33]. Maternal exposure to PM_2.5_ during pregnancy could indirectly affect fetal health by adversely affecting the health of the mother. Since PM_2.5_ has been associated with arterial narrowing [34,35], increased blood pressure[36-39], and impaired endothelial function[40], this exposure may impair the ability of the mother to deliver nutrients to the fetus. Alternatively, fine particles could directly affect the health of the infant, as fine particles are a mixture of different substances, many of the them toxic, such as metals, and can also have toxic organic matter, such as polycyclic aromatic hydrocarbons absorbed on their surface [41].

Our results are in agreement with the previous studies which analyzed the effect of exposure to PM_2.5_[10,29] and add weight to the conclusion that air pollutants negatively impact fetal development.

A major limitation of the present study is the spatial resolution of 10X10 km. While estimation conducted at a finer spatial resolution is preferable, PM_2.5_ is relatively homogeneous spatially [42], and so cells of this size probably capture most of the spatial variability in exposure to PM_2.5_, with the local traffic contribution captures by that separate covariate. Moreover this coarse resolution, combined with our use of a local traffic exposure variable allow us to focus on the effects of regional pollutants in this study. Another limitation of using a fixed 10x10km grid is the Modifiable areal unit problem (MAUP) which refers to the problem of information loss due to data aggregation [43], although MAUP is especially acute when observations are represented by simple point data [44] which is not the case in our study. It also should be noted that the study only used outdoor exposure concentrations, which may differ from indoor concentration and personal exposure but such data was unavailable.

As satellite remote sensing evolves and progresses, higher spatial resolution data (e.g 3x3km and 1x1 km) should become available in the next two years, which will further reduce exposure error. Such increased resolution should enable us to more precisely estimate daily exposures and how these vary across spatial locations.

## Conclusions

In summary, the presented study suggests that exposure to PM_2.5_ during pregnancy contributes to the risk of preterm birth and lower birth weight in infants.

## Competing interests

The authors declare that they have no competing interests.

## Authors' contributions

IK was the principal investigator responsible for design, conduct, analysis, interpretation of data and writing the manuscript. SJM was responsible for all GIS and spatial analysis work. WLR was responsible for design, data management and analysis. BAC participated as statistician and in the compilation and interpretation of the data and JS made contributions to conception, design, analysis of data and drafting the manuscript. All authors read and approved the final manuscript.
